# Genomic Insights into Neglected Orthobunyaviruses: Molecular Characterization and Phylogenetic Analysis

**DOI:** 10.3390/v17030406

**Published:** 2025-03-13

**Authors:** Safiétou Sankhe, Idrissa Dieng, Mouhamed Kane, Amadou Diallo, Ndeye Awa Ndiaye, Ndeye Marieme Top, Moussa Dia, Ousmane Faye, Amadou Alpha Sall, Oumar Faye, Pape Mbacke Sembene, Cheikh Loucoubar, Martin Faye, Moussa Moise Diagne

**Affiliations:** 1 Virology Department, Institut Pasteur de Dakar, Dakar BP 220, Senegal; safietou.sankhe@pasteur.sn (S.S.); idrissa.dieng@pasteur.sn (I.D.); mouhamed.kane-ext@pasteur.sn (M.K.); ndeyeawa0901@gmail.com (N.A.N.); moussa.dia@pasteur.sn (M.D.); ousmane.faye@pasteur.sn (O.F.); amadou.sall@pasteur.sn (A.A.S.); oumar.faye@pasteur.sn (O.F.); martin.faye@pasteur.sn (M.F.); 2 Animal Biology Department, Faculty of Sciences and Techniques, Cheikh Anta Diop University of Dakar, Dakar BP 5005, Senegal; mbacke.sembene@ucad.edu.sn; 3 Epidemiology, Clinical Research and Data Science Department, Institut Pasteur de Dakar, Dakar BP 220, Senegal; amadou.diallo@pasteur.sn (A.D.); ndeyemarieme.top@pasteur.sn (N.M.T.); cheikh.loucoubar@pasteur.sn (C.L.)

**Keywords:** neglected Orthobunyaviruses, genomic characterization, phylogenetics, zoonotic potential, vector-borne diseases

## Abstract

Orthobunyaviruses are a diverse group of segmented RNA viruses with significant but underexplored public and veterinary health implications. This study provides a genomic, phylogenetic, and ecological analysis of neglected Orthobunyaviruses using next-generation sequencing and computational predictions. We identified unique phylogenetic relationships, with Tanga virus forming a distinct lineage linked to zoonotic, human-associated, or non-vertebrate viruses across segments. GC content analysis revealed segment-specific patterns: higher GC content in the S segment suggests genomic stability and immune evasion, while lower GC content in the L segment reflects host-vector adaptation. Phylogenetic ties to well-characterized pathogenic viruses, such as Ilesha virus with Cache Valley virus and Bwamba virus with California encephalitis virus, indicate potential neurotropism. Ingwavuma virus clustered with Oropouche virus, suggesting risks of systemic febrile illnesses. Within the Simbu serogroup, Sango and Sabo viruses show teratogenic risks to livestock. Vector and host predictions implicate rodents, artiodactyls, and primates in Orthobunyavirus transmission, emphasizing complex ecological dynamics and zoonotic potential. These findings advance the understanding of Orthobunyavirus diversity, linking genomic features to pathogenicity and ecological adaptation, while providing a foundation for future surveillance and intervention strategies targeting these neglected viruses.

## 1. Introduction

Orthobunyaviruses (OBVs), the largest genus in the Peribunyavirus family with nearly 134 viral species [1], are arthropod-transmitted viruses characterized by a tripartite, negative-sense, and single-stranded RNA. These viruses pose significant threats to public and veterinary health due to their potential for zoonotic transmission and their ability to cause emerging and re-emerging diseases [2].

Despite their global importance, many OBVs are classified as neglected pathogens, because they remain historically under-researched, with limited knowledge about their epidemiology, pathogenesis, and impact on public health [3]. The OBV genome is segmented into three parts—S (small), M (medium), and L (large)—which encode various structural and non-structural proteins involved in viral replication, host immune evasion, and pathogenesis [4,5]. While at least 30 OBVs are known to cause diseases in humans [2,3], only a few have been studied extensively. Oropouche virus (OROV) is the second most frequent cause of acute febrile illness in tropical regions such as Central and South America [6,7,8], while La Crosse virus (LACV) is the leading cause of pediatric encephalitis in North America [9], infecting up to 300,000 persons each year, with severe cases involving central nervous system damage [10]. Similarly, California encephalitis virus (CEV), a member of the California serogroup causes brain inflammation [11]. Ngari Virus (NRIV) is pathogenic for both humans and animals, causing severe and often fatal hemorrhagic fever [12]. It was first isolated from humans in Senegal in 1993 [13] and has since been identified in several other African countries [14]. Cache valley virus (CVV) is a known teratogen in small ruminants, causing fetal death and severe malformations during epizootics in the United States [15] raising a major concern for public and agricultural health [16].

In addition to these relatively better known OBVs, several OBVs remain poorly studied but could have significant potential to impact human and animal health, these include Ilehsa virus (ILEV), Bwamba virus (BWAV), Ingwavuma virus (INGV), Simbu virus (SIMV), Sango Virus (SANV), Sabo virus (SABOV), M’Poko virus (MPOV), Botambi virus (BOTV), and Tanga virus (TANV). First identified in Nigeria, the Ilesha virus is suspected to cause hemorrhagic fevers and fatal meningoencephalitis [17,18]. During the 1980s, BWAV was among one of the most frequently reported arthropod-borne diseases in Africa [19], often mistaken for malaria [20] and causing death of mice by intracerebral or intranasal inoculation [21]. INGV, first isolated in 1970 from a pool of Culex vishnui complex mosquitoes in the Chiangmai Valley, Thailand, is now classified as Manzanilla OBV [22]. It is maintained in a pig–mosquito–bird transmission cycle, with human infection confirmed through virus isolation and the detection of neutralizing antibodies [23].

SIMV, SANV, and SABOV are part of the Simbu serogroup, a group of viruses known for their teratogenic effects. Viruses belonging to this serogroup are typically associated with abortions, stillbirths, congenital abnormalities, and significant economic losses in ruminants worldwide [24]. In 2019, SANV was identified and isolated in Israel from sera sampled from two symptomatic cows exhibiting fever, reduced milk production, and diarrhea [25]. Prior to this discovery, the only known strain (Ib An 5077) had been isolated in the 1960s in Ibidan, Nigeria, from *Culocoides* spp. [26].

MPOV, BOTV, and TANV are even less characterized, though the MPOV potential vector, *Culex perfuscus*, had been identified in 1966 in Bangui [27].

Understanding the genomic and ecological dynamics of neglected OBVs is critical for identifying emerging pathogens, assessing zoonotic risks, and mitigating their impact on public health and agriculture. Addressing these gaps requires targeted surveillance and intervention strategies informed by comprehensive genomic, phylogenetic, and ecological analyses. This study employs NGS and computational predictions to explore evolutionary relationships, genomic features, and potential reservoir and vector dynamics, offering insights into OBV pathogenicity and ecological adaptation. By characterizing 17 neglected OBV strains, we provide a foundation for future studies and targeted interventions, contributing to global public health efforts to address emerging infectious diseases and prevent zoonotic spillovers.

## 2. Materials and Methods

### 2.1. Archived Orthobunyavirus Strains Selection and Preparation

This study was carried out as part of the characterization of OBV strains archived in the biobank of the World Health Organization Collaborating Center for Arboviruses and Hemorrhagic Fever Viruses (CRORA) in the Institut Pasteur de Dakar (IPD). A total of seventeen viral strains for analysis, including four ILEV strains, five BWAV strains, one INGV strain, one SIMV strain, one SANV strain, one SABOV strain, one MPOV strain, one BOTV strain, and two TANV strains. A comprehensive epidemiological and virological overview of the nine viruses studied is presented in Table 1, while Table S1 provides detailed information on each strain, including its name, date and location of collection and isolation, and species of origin. All the viral strains were stored in a freeze-dried form within the IPD biobank and reactivated with 500 μL of 0.2% Bovine Serum Albumin (BSA) in PBS (1×) prior to inoculation in mice. A 1/10 dilution of the viral stock was prepared by mixing 100 μL of the stock with 900 μL of 0.2% Bovine Serum Albumin (BSA) in PBS. Each suckling mouse was then inoculated with 100 μL of this diluted viral stock, with groups of 10 mice used per strain. Clinical observations were recorded at various time points post-inoculation, focusing on signs such as recumbency, which appeared between days 3 and 7, depending on the viral strain. Brains were collected only from mice that succumbed to infection, and samples from the same strain were pooled for further analysis.

### 2.2. Sequencing and Bioinformatics Analyses

A total of seventeen viral strains archived at the IPD biobank were analyzed, encompassing a diverse set of OBV species. The strains were grouped as follows: four ILEV strains, five BWAV strains, one INGV strain, one SIMV strain, one SANV strain, one SABOV strain, one MPOV strain, one BOTV strain, and two TANV strains. Detailed information—including strain identification, associated virus group, and observed days post inoculation—is provided in Table S2. Following reactivation of freeze-dried viral stocks, groups of 10 suckling mice were inoculated with each viral strain. Observations indicated that the majority of strains exhibited clinical signs, particularly recumbency, between days 3 and 7 post inoculation. Among these, several strains consistently showed clinical effects at day 4, while others manifested signs earlier (day 3) or later (up to day 7). Such variability in the onset of clinical symptoms suggests differences in the virulence and/or replication kinetics among the different OBVs in the suckling mouse model. Based on these clinical observations, brains from mice inoculated with the same viral strain were pooled together for subsequent analysis.

RNA extraction was then performed on viral supernatants prepared from these pooled brains, using the QIAamp viral RNA mini-kit (Qiagen, Hilden, Germany) following the manufacturer’s recommendations before host ribosomal RNA (rRNA) depletion. Briefly, a first step of rRNA enzymatic depletion was carried out using an NEBNext rRNA Depletion Kit V2 (New England Biolabs, Hitchin, UK). cDNA synthesis was performed using the SuperScript IV Reverse Transcriptase Kit (Invitrogen, Thermo Fisher, Waltham, MA, USA), and sequencing libraries were produced using the Nextera-XT DNA Library Preparation Kit (Illumina, San Diego, CA, USA) following the manufacturer’s recommendations and as previously described [29]. Sequencing was performed on an Illumina NextSeq550 High output kit with 300 cycles cartridge using paired-end reads. Genome assembly was carried out using de novo assembly. After performing quality control and trimming using Trimmomatic v0.36 (Usadellab, Düsseldorf, Germany) [30] and the FastQC tool v0.11.5 (Bioinformatics Group, Babraham Institute, Cambridge, UK) [31], the reads were assembled de novo with SPADES v3.11.1 (Algorithmic Biology Lab, St Petersburg, Russia) [32]. The resulting contigs were analyzed using BLASTn v2.15 to identify the most closely matching reference sequence. The trimmed reads were subsequently mapped using the iVar v1.4.2 computational tool [33] to generate a consensus sequence, with a minimum read depth of 10X.

### 2.3. Phylogenetic Analysis

To perform genetics analyses and phylogenetic studies, we retrieved sequences for each genomic segment (S, M, L) from representative viral species across order (Hareavirales and Elliovirales), family (Peribunyaviridae), and genus (Orthobunyavirus) of the class Bunyaviricetes. The selected viruses are listed in Table S3. Phylogenetic trees for each segment were constructed using a maximum-likelihood (ML) implemented in IQ-TREE [34] with default parameters and 1000 bootstrap iterations for robust statistical support. ModelFinder (MF) performed automatic model selection based on the Bayesian Information Criterion (BIC) to enhance phylogenetic accuracy [35]. The resulting trees were visualized using FigTree v.1.4.3 [36].

### 2.4. Prediction of Reservoir Hosts, Vector and Arthropod-Borne Vectors

To predict the reservoir hosts, vectors, and arthropod-borne transmission potential of the studied OBVs, we used the Viral Host Predictor (VHP), a computational tool that leverages viral genetic sequences and machine learning algorithms to identify probable hosts and transmission dynamics [37]. VHP is particularly suited for identifying potential zoonotic viruses with the capacity to cross species barriers, including animal-to-human transmission. For Bunyaviruses, viral genome sequences corresponding to the S segment of each taxon were uploaded in FASTA format to the VHP platform [38].

For this study, VHP was applied to predict hosts and vectors for seven neglected OBVs: ILEV, BWAV, INGV, SIMV, SABOV, MPOV, and BOTV. Due to quality control (QC) limitations, SANV and TANV were excluded from the analysis. The model “Genomic biases + phylogenetic neighborhood” was selected due to its reliability in producing accurate results, mainly for groups of viruses with well-characterized transmission ecologies [37]. Predictions were based on the average output for each inference segment across all strains corresponding to the seven viral taxa, using the Bagged Prediction Strength (BPS) as a confidence metric, with a BPS value greater than 0.8 as high confidence. This approach ensured that the final predictions accounted for variations within individual taxa and provided a robust inference of reservoir hosts, vectors, and arthropod-borne transmission potential. Visualization was performed using an in-house R script [39] with the ggplot2 package [40].

## 3. Results

### 3.1. Orthobunyaviruses Sequencing Metrics

Bioinformatics metrics derived from the genomic analyses of various OBV strains—including GC content, sequencing coverage depth, and genome completeness—offered critical insights into the genomic diversity, structural characteristics, and sequencing quality of these viral genomes (Table S4).

**Figure 1:**
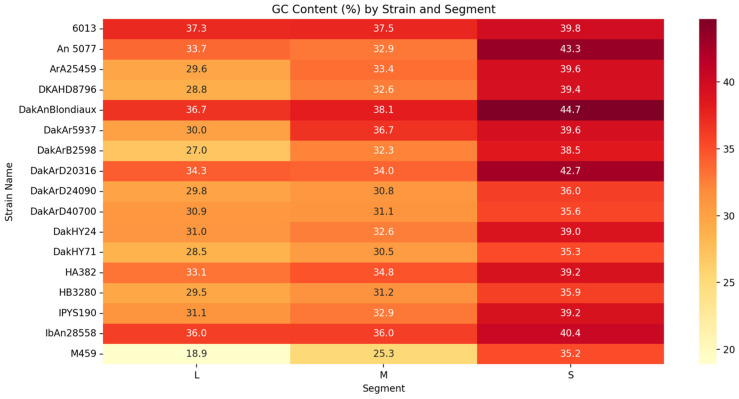
**Segment-specific GC content patterns across Orthobunyavirus strains.** Insights into genomic stability and evolutionary pressures, highlighting higher GC content in S segments and variability in L segments.

The GC content of OBV genomes revealed distinct segment-specific and strain-specific patterns, ranging from 28.8% to 44.7% (Figure 1). The S segment consistently exhibited the highest GC content, highlighting its likely role in genomic stability and immune evasion [41]. In contrast, the L segments displayed the lowest GC content, such as 18.9% in strain M459, suggesting adaptations to distinct functional or ecological roles. The strain-specific compositional differences may influence replication dynamics and host interactions. Strain-specific analyses revealed additional genomic variations. For instance, DakAnBlondiaux exhibited the highest GC content across all segments, suggesting potential adaptations to stable genomic functions. Conversely, strains like M459 displayed a unique low-GC profile in the L segment, which may reflect alternative evolutionary pressures. Coverage patterns also varied, with DKAHD8796 showing substantial variability in coverage depth and missing bases, while IPYS190 maintained more uniform metrics across its genome.

**Figure 2:**
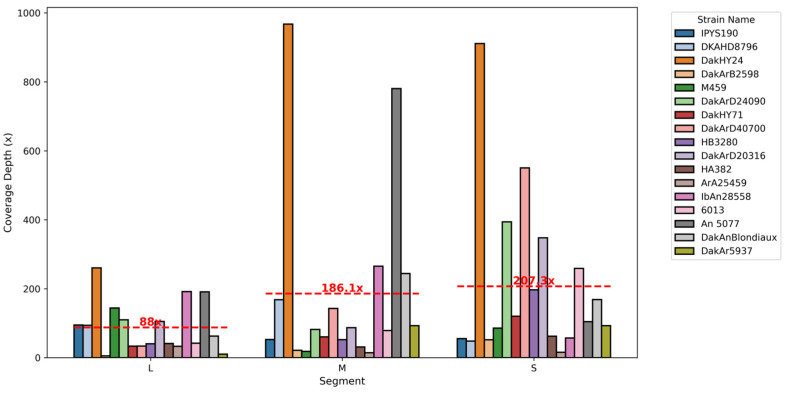
**Coverage depth of Orthobunyavirus genomic segments.** Variation in sequencing depth across strains and segments, emphasizing regions with higher sequencing quality and robustness.

Sequencing coverage depth varied significantly across strains and segments, ranging from 5.55X in the L segment of DakArB2598 to 967.52X in the M segment of DakHY24. On average, the S segments showed the highest coverage depths, as indicated by the annotated mean coverage lines in Figure 2. Strain-specific coverage trends were also observed, with DKAHD8796 demonstrating exceptional depth in the M segment, while IPYS190 exhibited consistent coverage across all segments. These patterns underline the robustness of the sequencing data and highlight genomic regions of particular interest for further study.

Genome completeness across strains was remarkably high, with most segments achieving >99.5% coverage. Missing bases were primarily observed in the L segments, such as 129 bases missing in M459 (Figure S1), reflecting inherent challenges in sequencing and assembling these regions. Despite these gaps, the sequencing quality remained robust (Table S2), supporting downstream analyses such as phylogenetics and ecological modeling.

These detailed analyses of GC content, coverage depth, and genome completeness collectively provide a solid foundation for understanding the evolutionary and structural complexities of OBV genomes. These metrics served as a critical resource for subsequent phylogenetic and ecological studies, enabling a deeper exploration of viral diversity, host interactions, and zoonotic potential.

### 3.2. Phylogenetic Relationships and Pathogenic Connections

To investigate the evolutionary relationships and genetic diversity of nine neglected OBVs, phylogenetic analyses were performed using genomic sequences from the L, M, and S segments. By integrating insights from both the neglected OBVs and better-characterized OBVs, we provide a comprehensive framework to understand the evolutionary dynamics driving viral diversity. Maximum-likelihood (ML) phylogenetic trees representative of the three viral segments were built using IQ-TREE with default parameters (General Time Reversible with Gamma-distributed rate variation) and 1000 bootstrap iterations.

**Figure 3:**
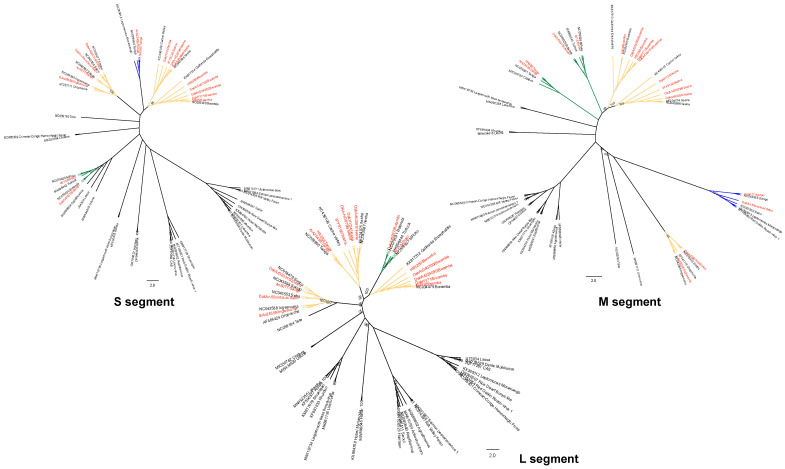
**Phylogenetic relationships of Orthobunyaviruses across genomic segments**. Maximum-likelihood phylogenetic trees for L, M, and S segments, revealing evolutionary ties to well-characterized pathogenic viruses. Each segment was assessed independently to explore genetic diversity and phylogenetic positioning. Bootstrap values indicate the confidence of clustering, with reference sequences from known Orthobunyaviruses included for comparison. Strains analyzed in this study are highlighted in red, showing their placement within the broader Orthobunyavirus phylogeny. Branch colors indicate virus associations: yellow for human-associated viruses, green for zoonotic viruses, and blue for non-vertebrate-associated viruses.

The phylogenetic analyses of the L, M, and S segments of Peribunyaviruses revealed broadly consistent evolutionary patterns across the three genomic regions, with minor lineage-specific differences (Figure 3). Almost all segments formed well-defined clades, with neglected OBVs showing significant phylogenetic connections to well-characterized OBVs that have established pathogenic profiles.

Phylogenetically, ILEV clusters closely with CVV, a well-characterized OBV associated with encephalitis and meningitis in humans. This close relationship suggests that ILEV may share neurotropic potential with CVV. The S segment of ILEV, encoding nucleocapsid proteins, is particularly significant, as it may facilitate replication in neural tissues. This connection raises concerns about the potential of ILEV to cause neurological diseases in humans or animals, emphasizing the need for further investigation into its pathogenic potential.

BWAV is phylogenetically close to CEV, a neurotropic OBV known for causing encephalitis in humans. Although BWAV is primarily associated with febrile illnesses and exhibits minimal meningeal involvement, its proximity to CEV suggests overlapping features, particularly in the M and S segments. These genomic similarities may hint at neurotropic properties that remain underexplored in BWAV, potentially expanding its known pathogenic profile.

INGV clusters with OROV, a well-documented OBV responsible for dengue-like syndromes in humans. INGV has already been implicated in human disease, with one infection confirmed through virus isolation. Its phylogenetic closeness to OROV suggests that INGV may also cause febrile or systemic illnesses, with the potential for broader human outbreaks under conducive ecological conditions.

MPOV and BOTV form a distinct cluster closely related to Turlock virus (TURV), a virus primarily associated with vector-host transmission cycles involving avian hosts and mosquito vectors. While TURV has not been linked to significant human pathogenicity, MPOV and BOTV may share its ecological niche, indicating potential for zoonotic spillover. Their unique clustering highlights the need for further study into their transmission dynamics and potential to infect human hosts.

SANV, SIMV, and SABOV cluster tightly within the Simbu serogroup, forming a distinct lineage with no close phylogenetic ties to other well-known OBVs. SIMV and SABOV are recognized for their teratogenic effects in livestock, including severe congenital malformations. Although SANV is less studied, its proximity within the Simbu cluster suggests that it may share these pathogenic traits, presenting risks to veterinary and agricultural health. Interestingly, in the M segment, SANV and SABOV are closer to non-vertebrate-associated viruses but are distinct from SIMV, highlighting unique features and suggesting particular ecological or functional adaptations.

TANV is distinct from all other OBVs, forming an independent lineage in the phylogenetic trees. This genetic divergence indicates unique ecological or functional adaptations. Importantly, TANV shows varying associations depending on the genomic segment analyzed. For the S segment, TANV clusters with non-vertebrate-associated viruses; for the M segment, it is more closely aligned with zoonotic viruses; and, for the L segment, its placement suggests ties with human-associated viruses. These unique segment-specific linkages underline its evolutionary and ecological diversity and suggest unexplored implications for disease ecology and potential zoonotic transmission. Limited pathogenic data necessitates further research to uncover the potential impact of TANV on public and animal health.

### 3.3. Prediction of Reservoir Hosts and Arthropod Vectors

**Figure 4:**
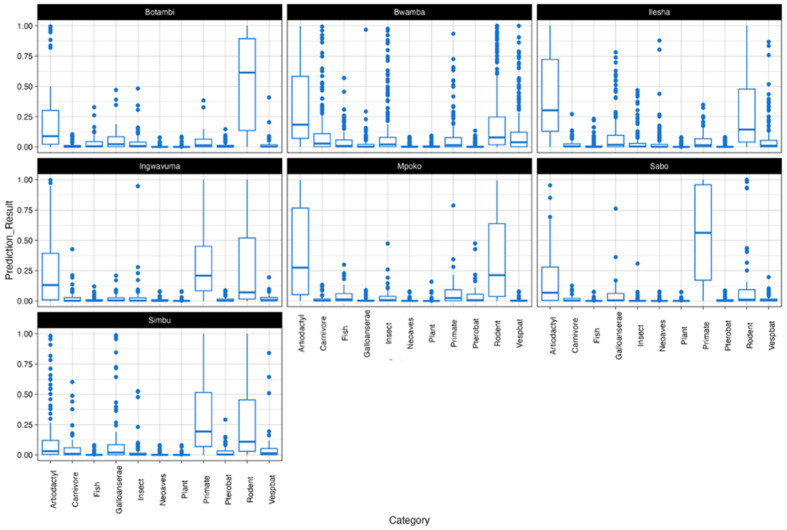
**Predicted reservoir hosts of neglected Orthobunyaviruses using viral host predictor (VHP).** High-confidence predictions of primary reservoir hosts, emphasizing roles of rodents, artiodactyls, and primates.

**Figure 5:**
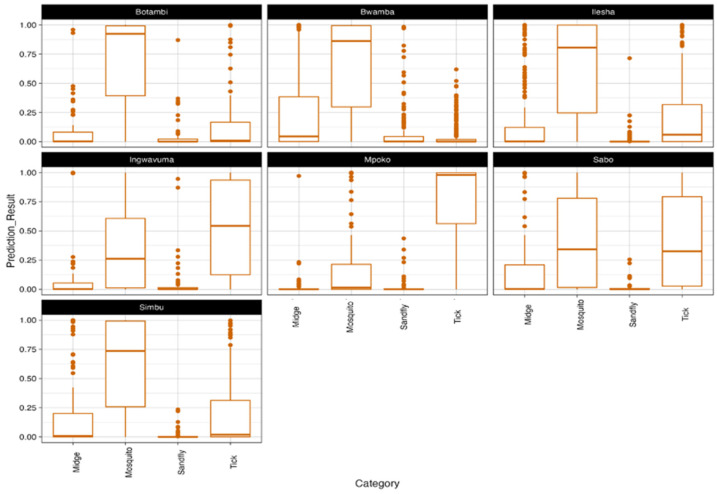
**Predicted arthropod vectors of neglected Orthobunyaviruses using viral host predictor (VHP)**. Predicted vector associations, highlighting mosquitoes as dominant vectors and complex dual transmission dynamics for certain strains.

Using the VHP, we analyzed the genomic sequences of seven neglected OBV strains (ILEV, BWAV, INGV, SIMV, SABOV, MPOV, and BOTV) to predict their potential reservoir hosts and arthropod vectors. While initial efforts included SANV and TANV, these taxa were ultimately excluded from the analysis due to QC constraints that affected their suitability for robust predictions. Predictions were based on the average output for each genomic segment (L, M, and S) across all strains within a given taxon, using the BPS metric as a confidence measure. BPS values above 0.8 were considered high confidence, ensuring that only strong associations were highlighted. Each individual prediction score corresponds to a specific genomic segment’s likelihood of association with a given host or vector category. The boxplots in Figure 4 and Figure 5 illustrate the distribution of the prediction scores, while the individual points represent the probability assigned to each genomic segment (L, M, S) of a given viral strain.

#### 3.3.1. Reservoir Hosts

Rodents emerged as the most strongly associated reservoir host across the majority of strains (Figure 4). Viruses such as BWAV and MPOV exhibited high-confidence predictions for rodent reservoirs, reflecting strong genomic signals aligning with this host group. Artiodactyls displayed strong support for viruses like BOTV and ILEV, highlighting their potential role in transmission cycles. Primates were also identified as key reservoirs, particularly for SABOV, which showed much higher confidence predictions for primates compared to rodents and artiodactyls. Predictions for plant reservoirs were consistently low across all strains, supporting their use as a negative control and demonstrating the specificity of the model in identifying biologically plausible hosts. Mixed or ambiguous predictions were observed for certain viruses. For instance, SIMV exhibited overlapping probabilities for both rodents and Pterobat (bats), suggesting possible bridge hosts or spillover dynamics.

#### 3.3.2. Arthropod Vectors

Mosquitoes emerged as the most strongly associated vectors for the majority of strains (Figure 5). Viruses such as BOTV, BWAV, ILEV, and SIMV exhibited high-confidence predictions for mosquito vectors, aligning with their known ecological roles in Orthobunyavirus transmission. Ticks were identified as secondary vectors, with MPOV showing a notable association. Moderate to high probabilities for tick vectors suggest their potential role in this strain’s transmission. Midges showed generally low prediction probabilities across most strains, except for minor associations with certain viruses. Similarly, predictions for sandflies were minimal, with almost no confidence across strains. Ambiguous or mixed predictions were observed for some viruses, reflecting ecological complexity. For instance, INGV exhibited overlapping probabilities for both mosquitoes and ticks, suggesting dual transmission ecology. Similar patterns, though to a lesser extent, were observed for ILEV and SABOV.

## 4. Discussion

This study presents a detailed molecular and phylogenetic characterization of ten neglected OBVs within the Peribunyavirus family. Utilizing NGS, computational prediction tools, and historical metadata, we provide insights into their genetic diversity, evolutionary relationships, potential reservoir hosts, and vector associations. These neglected OBVs pose a potential threat to public health and agricultural systems, necessitating increased awareness and further investigation into their pathogenic potential.

The GC content analysis revealed distinct segment-specific and strain-specific patterns, with the S segment consistently showing higher GC content. This suggests that the structural proteins encoded by the S segment might be under evolutionary pressure to maintain genomic stability and facilitate immune evasion. Strains like DakAnBlondiaux and An 5077 exhibit uniformly high GC content across segments, which might enhance their stability and adaptability. In contrast, strains such as M459 demonstrate notably low GC content, particularly in the L segment, possibly reflecting an alternative adaptation strategy. These findings highlight the potential link between GC content and pathogenicity, where higher GC content may enhance genome resilience, while lower GC content might confer flexibility in host adaptation [33].

The variability in GC content across OBV strains and segments highlights the influence of diverse evolutionary pressures. Selection for structural stability in GC-rich regions, particularly in the S segment, may ensure robust replication and immune evasion in hosts. Conversely, the lower GC content observed in the L segment may reflect mutational biases or adaptations to replication in vector environments. Host-specific codon usage biases, thermal adaptations, and ecological niches could contribute to this variability. Additionally, GC-rich regions, such as those observed in the S segment, may influence recombination dynamics by acting as stabilizing elements in some contexts, potentially reducing errors during strand exchange. This stability could facilitate the generation of recombinant strains with improved structural integrity, although the precise effects may vary depending on the genomic environment and recombination mechanisms involved [42]. Conversely, AT-rich regions, particularly in the L segment, may act as recombination hotspots due to their inherent structural flexibility. These dynamics underscore the dual role of GC content in balancing genome stability and adaptability, contributing to the diversification and ecological success of OBVs.

These findings underscore the interplay of mutational and selective pressures in shaping OBVs’ genomic evolution and highlight the adaptive strategies employed by these viruses to thrive in complex host-vector cycles. The relationship between GC content and recombination emphasizes its importance in influencing the evolutionary trajectories of OBVs, driving both their stability and their capacity for adaptive diversity.

The phylogenetic analysis of the L, M, and S genomic segments reveals that the nine neglected OBVs cluster with several well-characterized OBVs with known pathogenic profiles. This phylogenetic proximity suggests that the neglected viruses may exhibit similar pathogenic behaviors. ILEV closely clusters with CVV, which has been associated with encephalitis and meningitis in humans. The similarity of the S segment of ILEV to CVV, encoding nucleocapsid proteins implicated in replication in neural tissues, raises concerns about ILEV potential neurotropism. Historical metadata show that ILEV was isolated from human blood, supporting its direct involvement in human disease (Table 1). These findings underscore the need for further studies to determine if ILEV could be a cause of neurological disease in humans. BWAV shares a close evolutionary relationship with CEV, which is known for causing encephalitis in humans [43]. Although BWAV has primarily been associated with febrile illness, its phylogenetic closeness to CEV suggests underexplored neurotropic potential. BWAV was also isolated from mosquito species such as *Cellia gambiae* and *Diceromyia furcifer* (Table 1), which supports the viral host prediction of mosquitoes as the primary vectors. This strengthens the argument for further research into BWAV pathogenic profile and vector control efforts. INGV clusters closely with OROV, a known arbovirus associated with dengue-like illnesses [8]. Given INGV phylogenetic position and its isolation from mosquitoes, it is plausible that INGV might also cause similar systemic febrile illnesses in humans, especially under favorable ecological conditions.

SANV and SABOV cluster tightly with SIMV within the Simbu serogroup, consistent with their known teratogenic effects on livestock. The teratogenic impact of these viruses has significant agricultural implications, as they can cause congenital malformations in ruminants, affecting livestock productivity [44]. Historical metadata also indicate their isolation from livestock-associated areas, further supporting their potential to impact agriculture. This highlights the importance of surveillance and control measures to mitigate economic losses due to these viruses. Viruses such as MPOV and BOTV form distinct clusters closely related to TURV, suggesting ecological and transmission similarities, primarily involving avian hosts and mosquito vectors. Historical data reveal that MPOV was isolated from mosquitoes in regions with significant avian activity, which suggests a possible avian host involvement [45]. This supports their classification within a similar ecological niche to TURV, reinforcing the need for monitoring both avian populations and mosquito vectors to assess zoonotic risk. The phylogenetic independence of TANV highlights its unique evolutionary trajectory. The distinct phylogenetic placement, unlinked to other well-characterized OBVs, suggests it may have unique ecological niches and possibly novel mechanisms of host interaction, which remain largely unexplored.

Using the VHP tool, this study predicted the potential reservoir hosts and vectors for the studied OBVs, providing insights into their ecological interactions and the risk of zoonotic spillover. Rodents were consistently predicted as primary reservoirs for several viruses, including BWAV and MPOV. This aligns with the typical ecology of many OBVs, which utilize rodents as amplifying hosts. However, it is important to note that these predictions may reflect historical practices, as the viral strains were originally isolated using suckling mice before being archived.

Artiodactyls were identified as likely reservoirs for BOTV and ILEV, suggesting a potential for spillover events involving domesticated or wild ruminants. This emphasizes the need for increased surveillance of livestock, particularly in regions where these viruses are endemic. Primates were predicted as reservoirs for SABOV, highlighting potential zoonotic risks associated with human–primate interactions, especially in areas where primates serve as part of the viral transmission cycle. These findings highlight the role of reservoirs like rodents, artiodactyls, and primates in OBVs’ transmission, emphasizing the need for a One Health approach to mitigate zoonotic spillover.

Regarding vectors, mosquitoes emerged as the predominant predicted vectors for most of the neglected OBVs, indicating their significant role in the transmission cycle of these viruses. The high-confidence predictions for mosquito vectors highlight the need for vector control strategies to mitigate human exposure to these viruses. Historical evidence, including the isolation of BWAV, MPOV, and BOTV from mosquitoes, aligns with these predictions and underscores the importance of integrated vector management. Ambiguous or mixed vector predictions for some viruses, such as SIMV and INGV, indicate possible associations with both rodents and bats, indicating spillover dynamics. This dual association suggests a complex ecological interaction that requires focused studies on cross-species transmission to understand possible zoonotic risks.

This study highlights the urgent need for enhanced surveillance and research into neglected OBVs, given their genetic similarity to well-known pathogenic viruses and their potential for zoonotic transmission. The predicted reservoir hosts and vectors align well with known OBV ecology, suggesting that these neglected viruses may already be circulating among wildlife, livestock, and vectors, with underappreciated impacts on public and veterinary health. The clustering of viruses like ILEV, BWAV, and INGV with known neurotropic and systemic pathogenic OBVs raises concerns about their potential to cause human and animal diseases. In particular, the neurotropic potential of ILEV and BWAV, as suggested by their phylogenetic relationships and isolation from human hosts, underscores the importance of neurological surveillance, particularly in regions where these viruses are endemic. In agricultural systems, viruses such as SIMV, SANV, and SABOV, which are closely related to known teratogenic viruses [24], present risks to livestock productivity. The economic impact of congenital malformations in ruminants can be substantial, and proactive measures, such as vaccination and vector control, could help mitigate these losses.

To better understand the public and veterinary health impact of these neglected OBVs, we recommend the following: (i) Pathogenicity studies are necessary to elucidate the pathogenic potential of these viruses, particularly those closely related to neurotropic and systemic OBVs. (ii) Comprehensive ecological studies investigating the natural reservoirs and vectors of these viruses will provide crucial insights into their transmission dynamics and potential for spillover. (iii) Enhanced arbovirus surveillance, particularly in endemic regions, is crucial for early detection and response to potential outbreaks, supported by the deployment of molecular diagnostic tools for rapid pathogen identification. (iv) Integrating genomic characterization into routine surveillance could facilitate the identification of emerging viral threats.

## 5. Conclusions

The neglected OBVs characterized in this study share significant genetic relationships with well-characterized OBVs that are known to cause severe diseases in humans and animals. Genomic analysis highlights segment-specific variability in GC content, reflecting evolutionary pressures and functional adaptations. The higher GC content in the S segment suggests stability and immune evasion, while lower GC content in the L segment indicates mutational biases and host-vector adaptations. The integration of phylogenetic analysis, computational predictions, and historical metadata provides compelling evidence for the zoonotic potential of several of these viruses.

While this study contributes to our understanding of OBVs, further research is needed to confirm their pathogenic potential, ecological interactions, and zoonotic risks. These findings underscore the importance of continued surveillance and multidisciplinary approaches to better characterize and mitigate the potential threats posed by these under-researched viruses.

## Figures and Tables

**Table 1:** Comprehensive overview of epidemiological and vector associations of neglected Orthobunyaviruses.

Virus Name	Viral Species	First Identification (Place and Year)	Reported Human Case	Geographical Distribution	Vectors/Isolation Species	Clinical Manifestations
Bwamba Virus (BWAV)	*Orthobunyavirus bwambaense*	Uganda in 1937 [20]	Yes	Uganda, Central African Republic, South Africa, Cameroun, Senegal	*Anopheles gambiae* *Anopheles funestus*	Exanthem with meningeal involvement, fever, headache, arthralgia, diarrhea, body rash.
Ilesha Virus (ILEV)	*Orthobunyavirus ileshaense*	Nigeria in 1957 [28]	Yes	Central African Republic, Cameroun, Madagascar, Senegal	*Anopheles gambiae*	Fatal meningoencephalitis, hemorrhagic fever
Ingwavuma Virus (INGV)	*Orthobunyavirus ingwavumaense*	Thailande in 1970 [22]	Yes	Indonesia, India, Thailand, Nigeria	*Culex vishnui* *Hyphanturgus ocularius*	Febrile illnesses
Simbu Virus (SIMV)	*Orthobunyavirus simbuense*	Nigeria in 1964–1969 [26]	No	South Africa, Nigeria, Israel	Mosquito and midge	Unknown
Sango Virus (SANV)	*Orthobunyavirus sangoense*	Nigeria in 1965 [26]	No	Nigeria, Israel	*Culocoides* spp. Blood cattle	Unknown
Sabo Virus (SABOV)	*Orthobunyavirus saboense*	Nigeria in 1966 [26]	No	Nigeria, Central African Republic	Blood goat	Unknown
M’Poko Virus (MPOV)	*Orthobunyavirus mpokoense*	Central African Republic in 1966 [27]	No	Central African Republic, Senegal	*Culex pruina* *Culex weschei* *Culex perfuscus*)	Unknown
Botambi Virus (BOTV)	*Orthobunyavirus botambiense*	Central African Republic in 1968 [27]	No	Central African Republic, Cote d’Ivoire	*Culex guiarti*	Unknown
Tanga Virus (TANV)	*Orthobunyavirus tangaense*	Tanzania in 1962 [27]	No	Tanzania, Burkina Faso, Cote d’Ivoire	*Anopheles funestus*	Unknown

## Data Availability

The genomic sequences obtained in this study have been deposited in the NCBI GenBank repository under the accession numbers provided directly within the manuscript.

## Supplementary Materials

The following supporting information can be downloaded at: https://www.mdpi.com/article/10.3390/v17030406/s1, Figure S1: Distribution of Missing Bases Across Segments (L, M, S) by Orthobunyavirus Strains; Table S1: Summary of Metadata for the Studied Orthobunyavirus Strains; Table S2: Summary of Virus Strains and Timepoints of Mortality in Inoculated Mice; Table S3: Representative Viral Species and Genomic Segments Retrieved for Genetic and Phylogenetic Analyses; Table S4: Bioinformatics Metrics from Genomic Analyses of Orthobunyavirus Strains.
