# Supplementary material for: Genomic Insights into Neglected Orthobunyaviruses: Molecular Characterization and Phylogenetic Analysis
Source: Viruses. 2025 Mar 13;17(3):406. doi: 10.3390/v17030406 (PMC11945402; doi:10.3390/v17030406)
Supplement: Supplementary file 1 [file viruses-17-00406-s001.zip › Table S1-3450473.pdf]

Table S1. Summary of Metadata for the Studied Orthobunyavirus Strains.

Virus	Strain	Collection date (Year-Month-Day)	Collection site	Isolation date (Year-Month-Day)	Isolation location	Host
Ilesha Virus (ILEV)	Dak HY 24	1970-02-16	Yaoundé - Cameroun	1970-03-16	Centre Pasteur du Cameroun	Human Blood from 18 years old Woman
	Dak Ar B2598	1970-03-06	Bangui - RCA	1970-03-23	Institut Pasteur de Bangui	Mosquito / Cellia gambiae s. l.
	IP YS 190	1964-08-19	Yaoundé - Cameroun	1965-11-24	Centre Pasteur du Cameroun	Human Serum
	DKA HD 8796	1969-08-29	Bandia - Sénégal	1969-09-07	Institut Pasteur de Dakar	Human Blood from 17 years old African
Bwamba Virus (BWAV)	Dak Ar D 40700	1984-07-24	Fadiga - Kédougou - Sénégal	1984-11-20	Institut Pasteur de Dakar	Mosquito / Diceromyia furcifer
	HB 3280	1965-04-19	Bangui - RCA	1967-11-23	Institut Pasteur de Bangui	Human Serum
	M459	1937-10-15	Entebbe - Uganda	1937-12-15	Entebbe	Human Serum
	Dak ArD 24090	1976-06-22	Kédougou - Sénégal	1976-07-20	Institut Pasteur de Dakar	Mosquito / Cellia gambiae s. l.
	Dak HY 71	1974-01-20	Yaoundé - Cameroun	1974-03-20	Centre Pasteur du Cameroun	Human Serum
Ingwavuma Virus (INGV)	Ib An 28558	1968-07-09	Ibadan - Nigéria	1969-07-10	Institut Pasteur de Dakar	Plesiositagra cucullatus
Simbu Virus (SIMV)	Dak ArD 20316	1974-09-24	Kédougou - Sénégal	1974-10-22	Institut Pasteur de Dakar	Mosquito / Aedimorphus dalzieli
Sango Virus (SANV)	An 5077	1965-04-26	Ibadan - Nigéria	1965-05-26	Nigéria	Mammalian / Cattle
Sabo Virus (SABOV)	Dak An Blondiaux	1983-08-23	Bangui - RCA	1983-09-23	Institut Pasteur de Bangui	Mammalian / Sheep
M'Poko Virus (MPOV)	6013	1990-06-18	Dakar - Bango	1991-01-11	Institut Pasteur de Dakar	Mosquito
Botambi Virus (BOTV)	Dak Ar 5937	1982-08-14	Abidjan - Cote d'ivoire	1982-09-14	Institut Pasteur de Côte d’Ivoire	Mosquito / Culex
Tanga Virus (TANV)	HA 382	Undetermined	Burkina Faso	1984-02-02	Institut Pasteur de Côte d’Ivoire	Human Blood
	Ar A 25459	1989-10-01	Dezidougou - Cote d'Ivoire	1991-02-13	Institut Pasteur de Côte d’Ivoire	Mosquito / Cellia funestus
