# Supplementary figures and images for: Genomic Insights into Neglected Orthobunyaviruses: Molecular Characterization and Phylogenetic Analysis
Source: Viruses. 2025 Mar 13;17(3):406. doi: 10.3390/v17030406 (PMC11945402; doi:10.3390/v17030406)

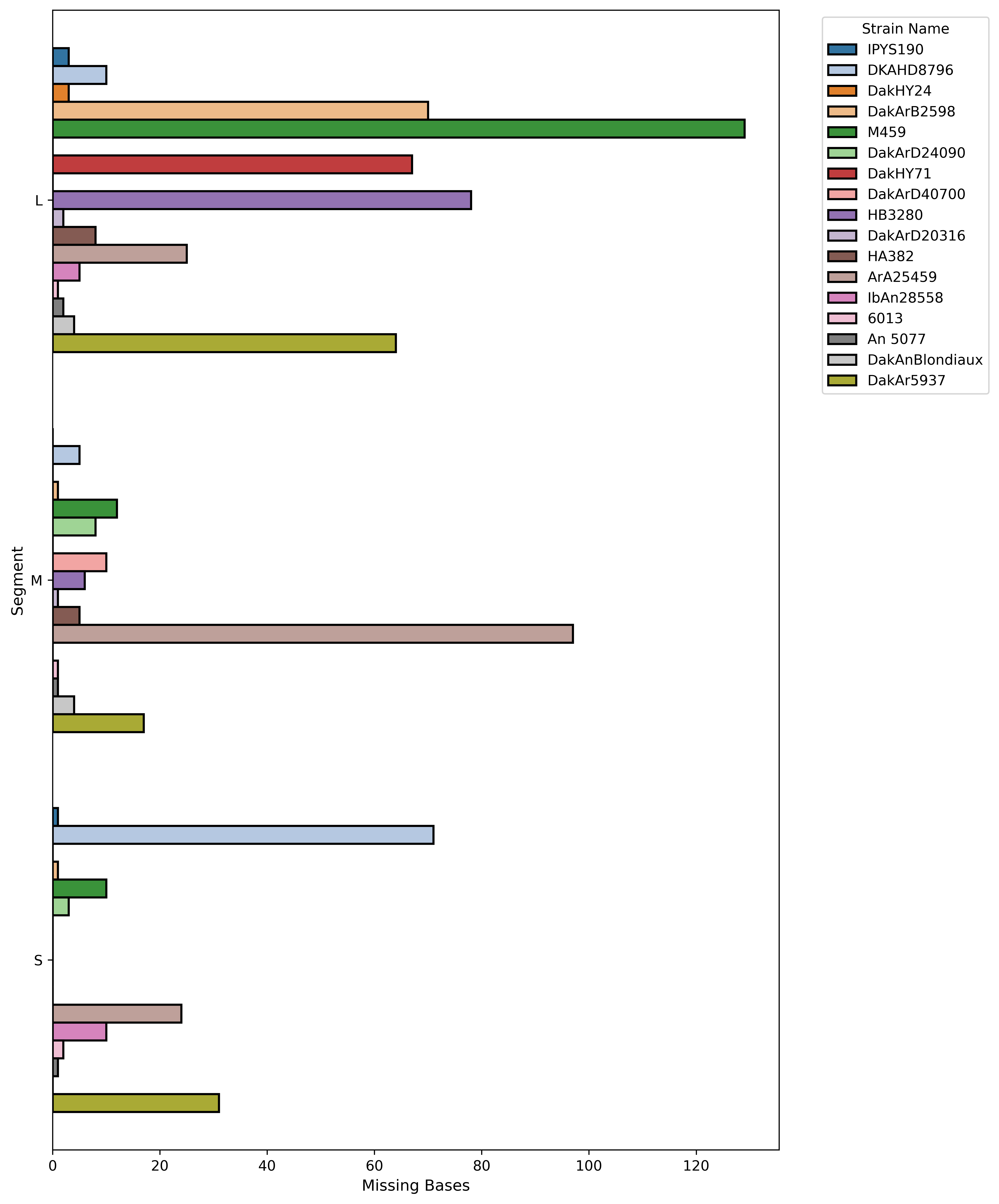

Supplement: Supplementary file 1 [file viruses-17-00406-s001.zip › Figure S1-3450473.png]
